# Left Atrioventricular Transvalvular Pressure Gradients Derived from Intraoperative and Postoperative Echocardiograms following Atrioventricular Septal Defect Repair

**DOI:** 10.3390/diagnostics13050957

**Published:** 2023-03-02

**Authors:** Maximilian Bamberg, Mark Simon, Andrea Bandini, Julia Kelley Hahn, Christian Schlensak, Vanya Icheva, Michael Hofbeck, Peter Rosenberger, Harry Magunia, Marius Keller

**Affiliations:** 1 Department of Anesthesiology and Intensive Care Medicine, University Hospital Tuebingen, Eberhard-Karls-University, Hoppe-Seyler-Strasse 3, 72076 Tuebingen, Germany; 2 Department of Thoracic and Cardiovascular Surgery, University Hospital Tuebingen, Eberhard-Karls-University, Hoppe-Seyler-Strasse 3, 72076 Tuebingen, Germany; 3 Department of Pediatric Cardiology, Pulmonology and Intensive Care Medicine, University Hospital Tuebingen, Eberhard-Karls-University, Hoppe-Seyler-Strasse 1, 72076 Tuebingen, Germany

**Keywords:** atrioventricular septal defect, congenital heart disease, cardiac surgery, echocardiography, left atrioventricular valve

## Abstract

Background: Left atrioventricular valve (LAVV) stenosis following an atrioventricular septal defect (AVSD) repair is a rare but potentially life-threatening complication. While echocardiographic quantification of diastolic transvalvular pressure gradients is paramount in the evaluation of a newly corrected valve function, it is hypothesized that these measured gradients are overestimated immediately following a cardiopulmonary bypass (CPB) due to the altered hemodynamics when compared to postoperative valve assessments using awake transthoracic echocardiography (TTE) upon recovery after surgery. Methods: Out of the 72 patients screened for inclusion at a tertiary center, 39 patients undergoing an AVSD repair with both intraoperative transesophageal echocardiograms (TEE, performed immediately after a CPB) and an awake TTE (performed prior to hospital discharge) were retrospectively selected. The mean (MPGs) and peak pressure gradients (PPGs) were quantified using a Doppler echocardiography and other measures of interest were recorded (e.g., a non-invasive surrogate of the cardiac output and index (CI), left ventricular ejection fraction, blood pressures and airway pressures). The variables were analyzed using the paired Student’s t-tests and Spearman’s correlation coefficients. Results: The MPGs were significantly higher in the intraoperative measurements when compared to the awake TTE (3.0 ± 1.2 vs. 2.3 ± 1.1 mmHg; *p* < 0.01); however, the PPGs did not significantly differ (6.6 ± 2.7 vs. 5.7 ± 2.8 mmHg; *p* = 0.06). Although the assessed intraoperative heart rates (HRs) were also higher (132 ± 17 vs. 114 ± 21 bpm; *p* < 0.001), there was no correlation found between the MPG and the HR, or any other parameter of interest, at either time-point. In a further analysis, a moderate to strong correlation was observed in the linear relationship between the CI and the MPG (r = 0.60; *p* < 0.001). During the in-hospital follow-up period, no patients died or required an intervention due to LAVV stenosis. Conclusions: The Doppler-based quantification of diastolic transvalvular LAVV mean pressure gradients using intraoperative transesophageal echocardiography seems to be prone to overestimation due to altered hemodynamics immediately after an AVSD repair. Thus, the current hemodynamic state should be taken into consideration during the intraoperative interpretation of these gradients.

## 1. Introduction

**Figure 1:**
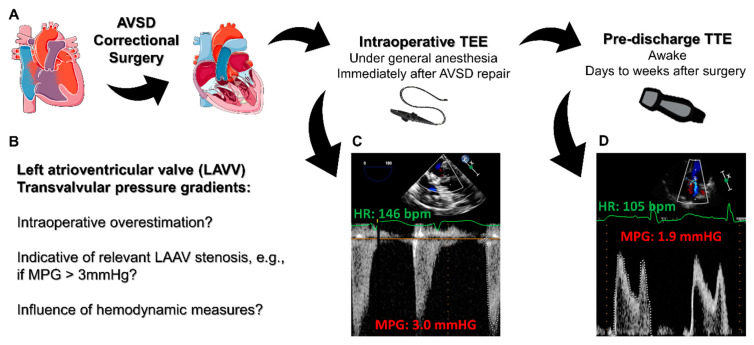
Graphical overview of the study’s design, underlying questions and exemplary findings. The patient cohort consisted of retrospectively selected pediatric patients undergoing surgical repair of a congenital atrioventricular septal defect (AVSD) whose transvalvular pressure gradients of the left atrioventricular valve (LAVV) were quantified with an intraoperative transesophageal echocardiography (TEE) immediately after an AVSD repair and an awake transthoracic echocardiography (TTE) prior to hospital discharge (**A**). The study was conducted to facilitate the interpretation of LAVV transvalvular pressure gradients following an AVSD at the two time-points, focusing on the detection of relevant postoperative LAVV stenosis and the role of the hemodynamic state (**B**). Representative pulsed-wave Doppler-based quantification of a patient using intraoperative TEE (**C**) and pre-discharge TTE (**D**), illustrating E/A wave fusion with higher heart rates. MPG = mean pressure gradient.

An atrioventricular septal defect (AVSD) is a rare congenital heart anomaly with potentially severe limitations for physical capacity, which is often associated with genetic disorders, other cardiac defects and extracardiac manifestations [1]. As patient prognosis can be significantly limited, an early surgical AVSD repair in a cardiopulmonary bypass (CPB) at a young age is the treatment of choice in most patients, which leads to a dramatic improvement in survival [2,3]. Awake transthoracic echocardiography (TTE) plays a pivotal role in initially assessing the underlying type and degree of an AVSD, facilitating essential diagnostic information necessary for evaluating the patient for surgical repair [4,5]. An intraoperative transesophageal echocardiography (TEE), on the other hand, is a useful tool to not only confirm the preoperative TTE measurements but to also detect immediate complications following a surgical AVSD repair [6,7]. An atrioventricular cleft closure and reconstruction of the left atrioventricular valve (LAVV) can potentially result in relevant narrowing of the LAVV or its annulus, causing iatrogenic LAVV stenosis and subsequent elevated left atrial pressures [8]. While postoperative LAVV stenosis is a rare complication when compared to LAVV regurgitation, it can have detrimental effects on a patients’ health and usually requires an immediate return to the CPB for surgical revision [9,10,11]. To date, data regarding the appropriate interpretation of the intraoperative diastolic LAVV transvalvular pressure gradients immediately after an AVSD surgical repair are lacking, and it remains unclear if and to what extent these gradients might be influenced by hemodynamic measures. Furthermore, the relationship between the intraoperative TEE-derived LAVV gradients and measurements performed using an awake TTE is unknown. The present study, therefore, investigates this relationship in a retrospectively selected pediatric cohort, hypothesizing that LAVV transvalvular pressure gradients measured with an intraoperative TEE immediately after an AVSD repair are overestimated when compared to awake postoperative TTE measurements prior to hospital discharge (Figure 1A,B).

## 2. Materials and Methods

### 2.1. Ethical Approval, Study Design and Patient Selection

This study was conducted in accordance with the Declaration of Helsinki and approved by the Ethics Committee of the University of Tuebingen (project number # IRB 773/2019BO2 with an amendment on 29 April 2022). According to German privacy regulations, anonymous retrospective analysis, including the handling of clinically acquired data does not require informed consent from individual patients. For this single-center retrospective cohort study, patients undergoing a surgical AVSD repair, including atrial and ventricular cleft closure between May 2015 and June 2021 were identified within the digital clinical database system. Only patients with both intraoperative TEEs and pre-discharge TTEs, allowing for the quantification of the below mentioned echocardiographic measures, were included in the final analysis.

### 2.2. Anesthesia, Surgical Technique and Cardiopulmonary Bypass

General anesthesia with endotracheal intubation and positive pressure ventilation were applied either as total intravenous anesthesia (using a combination of midazolam and fentanyl) or balanced anesthesia (with sevoflurane and sufentanil). All infants received advanced hemodynamic monitoring with arterial cannulation and central venous pressure monitoring. 

Surgical repair of the AVSD using the double patch technique and cleft closure was routinely performed via sternotomy with the use of CPB and aortic cross clamping. Prior to the completion of the surgery, an appropriate minimum LAVV orifice area was routinely evaluated using a Boulito probe standardized to the patients’ body surface area (BSA), and the water probe method was used to exclude any significant LAVV regurgitation.

### 2.3. Echocardiography

Intraoperative TEE was performed after separation from the CPB and the establishment of a steady hemodynamic state by specially trained cardiac anesthesiologists and congenital cardiologists using commercially available probes (S7-3t or S8-3t TEE probes, Philips Healthcare, Inc., Andover, MA, USA). Postoperative echocardiography was performed around the day of discharge by congenital cardiologists using commercially available probes (S8-3 or S12-4 sector probes, Philips Healthcare, Inc., Andover, MA, USA). All echocardiographic studies were stored in the institutional digital database system (IntelliSpace Cardiovascular, Philips Healthcare, Inc., Andover, MA, USA Cardiovascular) and the offline measurements were performed within the software’s interface.

At least two tracings of the diastolic pulsed-wave or continuous-wave Doppler spectra over the LAVV in the apical/mid-esophageal four-chamber view yielded the following: mean pressure gradient (MPG), peak pressure gradient (PPG) and velocity-time integral (VTI). At the time of the measurements, the heart rate (HR) was recorded. With the help of the color-coded Doppler recordings in the same view, the diameter of the LAVV orifice was measured. A non-invasive surrogate of the cardiac output (CO) was calculated according to the following formula: CO [L/min) = (LAVV diameter [mm]/2)² × 3.141 × VTI [mm] × HR/1,000,000. The cardiac index (CI) was calculated as follows: CI [L/min/m²] = CO [L/min]/BSA [m²]. The left ventricular volumes and ejection fractions (LVEF) were quantified using the uniplane or biplane Simpson’s method.

### 2.4. Clinical and Follow-Up Data

The clinical data were extracted from the electronic patient records within the institutional databases and included the baseline demographic characteristics, preoperative echocardiographic parameters, symptoms of heart failure and comorbidities. The patients’ BSA was calculated using the Mosteller method: BSA [m²] = (height [cm] × weight [kg]/3600)^0.5^. The exact surgical intervention, CPB times and intraoperative hemodynamics were extracted from the clinical database, including the mean arterial pressure (MAP), central venous pressure (CVP) and airway pressures, at the time of the intraoperative TEE. The Vasoactive-Inotropic Score (VIS) was calculated as previously described [12].

The follow-up data included the postoperative length of stay in the intensive care unit and the hospital, the duration of the postoperative mechanical ventilation and any adverse events within the primary hospital stay. Long-term follow-up data was recorded if the patients were admitted to our center within the study period.

### 2.5. Statistical Analysis

Normally distributed continuous variables are presented as the mean ± standard deviation, while nonnormally distributed continuous variables are presented as the median (interquartile range). The categorical variables are reported as absolute numbers and percentages. Comparisons of the parameters between the two echocardiographic time-points were performed using the paired Student’s t-tests for normally distributed continuous variables, Wilcoxon matched-pairs signed rank tests for nonnormally distributed continuous variables and Chi-square tests to compare the proportions. Spearman’s correlation coefficient (r) was used to assess the degree of linear relationship between two non-normally distributed variables and Pearson’s r was used if both samples were normally distributed. The degree of correlation was defined as follows: “none” for r from 0 to ±0.2, “weak” for r from ±0.2 to ±0.4, “moderate” for r from ±0.4 to ±0.6, “strong” for r from ±0.6 to ±0.8 and “very strong” for r from ±0.8 to ±1. *p*-values of < 0.05 were regarded as significant for most analyses. For extensive multiple testing (Spearman’s correlation matrices), the significance level α was corrected according to the Bonferroni method. In the figures, significant p-values are highlighted as * *p* < 0.05, ** *p* < 0.01 and *** *p* < 0.001. Prism (GraphPad Software Inc., San Diego, CA, USA) and MedCalc (MedCalc Software Ltd., Ostend, Belgium) were used for statistical calculations and data presentation.

## 3. Results

### 3.1. Clinical and Surgical Characteristics

Within the study period, 72 patients underwent an AVSD repair at our center. Among this group of patients, 29 (40%) patients did not receive Doppler measurements over the LAVV prior to discharge and four (6%) patients’ image quality was insufficient in either of the performed echocardiograms, resulting in the inclusion of 39 patients in the final analysis. Baseline demographic and clinical characteristics are displayed in Table 1. The majority of the cohort suffered from complete AVSD (87%), while partial (10%) and transitional (3%) forms were less frequent. Left and right atrioventricular valve regurgitation was present in 82% and 72% of the patients, respectively. Only a small fraction showed an impaired left (10%) or right ventricular (10%) systolic function prior to surgery, with 82% (32 out of 39) presenting with symptoms of heart failure and 51% with pulmonary hypertension. Notably, 64% of the patients had trisomy 21. Surgical and intraoperative data are listed in Table 2. LAVV reconstruction was performed in 62% of the cases, and 13% of the total cohort presented with an absence of echocardiographic signs of LAVV regurgitation after an AVSD repair. The intraoperative vasoactive-inotropic substance of choice was milrinone (used in 90% of the patients), while norepinephrine (33%) and epinephrine (15%) were used less frequently.

### 3.2. Echocardiographic Data

**Figure 2:**
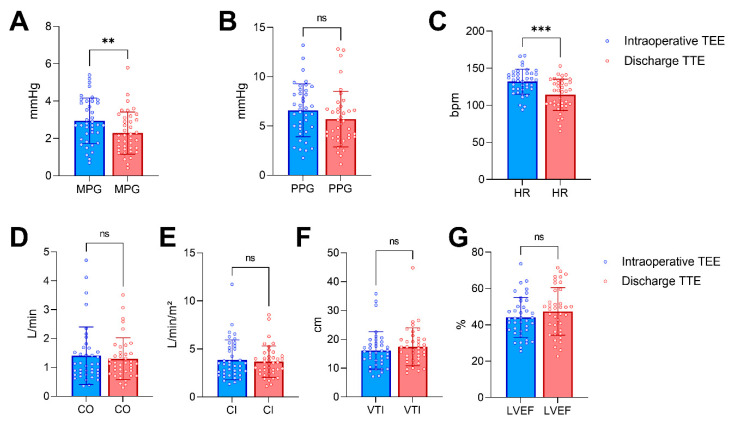
Intraoperative and pre-discharge echocardiographic and hemodynamic variables. Comparison of the left atrioventricular valve (LAVV) mean pressure gradient (MPG, **A**), LAVV peak pressure gradient (PPG, **B**), heart rate (HR, **C**), cardiac output (CO, **D**), cardiac index (CI, **E**), LAVV velocity-time integral (VTI, **F**) and left ventricular ejection fraction (LVEF, **G**) derived from the intraoperative transesophageal echocardiography (TEE, blue color) and awake transthoracic echocardiography (TTE, red color) prior to discharge. Bars and antennas represent means and standard deviations, respectively. Comparisons were performed with the paired Student’s tests or the Wilcoxon matched-pairs signed rank tests. ** = *p* < 0.01, *** = *p* < 0.001, ns = not significant.

**Figure 3:**
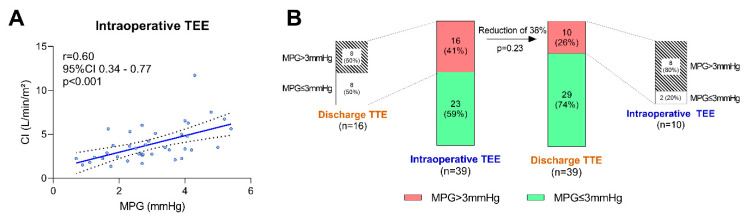
Characteristics of left atrioventricular valve (LAVV) mean pressure gradient (MPG) quantification following an AVSD repair using intraoperative transesophageal echocardiography (TEE). Non-invasively quantified cardiac index (CI) as a surrogate for the hemodynamic state shows a moderate-to-strong linear correlation with LAVV MPG at the intraoperative time-point (**A**). Proportions of patients with LAVV MPG above or below/equal to 3 mmHg quantified with intraoperative TEE and pre-discharge awake transthoracic echocardiography (TTE), respectively (**B**). 95%CI = 95% confidence interval, r = (Pearson’s) correlation coefficient.

The pulsed-wave Doppler quantification of the LAVV gradients was performed in 34 patients (87%), while the remaining patients were analyzed with the continuous-wave Doppler. The Doppler measurements were compared concomitantly (PW with PW, or CW with CW when PW was not available in both TEE and TTE; never CW with PW or vice versa). Table 3 depicts the results of the echocardiographic measurements performed intraoperatively via TEE and postoperatively via TTE prior to discharge. Representative measurements are displayed in Figure 1C,D. As hypothesized, the LAVV mean pressure gradients were significantly higher intraoperatively when compared with the discharge values (mean difference 0.7 mmHg, 95% confidence interval 0.3–1.1 mmHg, *p* < 0.01; Figure 2A), while the PPGs did not differ significantly between the two time-points (Figure 2B). The HRs at the time of the intraoperative TEE were significantly higher than those at the discharge TTE (mean difference 18 bpm, 95% confidence interval 11–25 bpm, *p* < 0.001; Figure 2C). The CO, CI, LAVV VTI and LVEF, however, did not change significantly between the two echocardiographic studies (Figure 2D–G). The correlation matrices were calculated to evaluate potential associations between the echocardiographic measures of LAVV gradients and the hemodynamics (α = 0.001, Figure S1). Notably, there was a moderate to strong correlation (r = 0.60, *p* < 0.001) between the non-invasively determined CI and the LAVV MPG immediately after weaning from the CPB (Figure 3A), while the correlation between the CI and the MPG using the awake TTE prior to discharge was not significant (r = 0.39, *p* = 0.02, Figure S2A). The change in the MPG between the intraoperative TEE and the pre-discharge TTE (∆MPG) further showed no correlation with the intraoperative HR (r = −0.07, *p* = 0.67, Figure S2B) and the intraoperative CI (r = 0.38, *p* = 0.02, Figure S2C), respectively. No meaningful correlations were present between the LAVV transvalvular pressure gradients and the remaining measures, including the extent of the mechanical ventilation (airway pressures) and the hemodynamic condition (HR, MAP, CVP, VIS). The proportion of patients with a LAVV MPG of ≥3 mmHg was 41% (16/39) intraoperatively and 26% (10/29) prior to discharge (*p* = 0.23, Figure 3B). In a further analysis, only 50% (8 out of 16) of patients with an intraoperative elevated MPGs of ≥3 mmHg also presented with an MPG above 3 mmHg prior to discharge, while 80% (8/10) of the patients with an elevated MPG at discharge also previously showed an elevated MPG ≥3 mmHg in the intraoperative TEE measurements.

### 3.3. Outcome

The data collected on the postoperative course of the studied patients is summarized in Table 4. All patients were discharged alive. One patient underwent successful cardiopulmonary resuscitation due to postoperative asystole. One patient with prolonged low cardiac output syndrome recovered after receiving inotropic support with milrinone and epinephrine. Of the five patients with pericardial or pleural effusion, one required thoracotomy and two required chest drains. No external defibrillation or pacemaker implantations were required to treat the remaining adverse arrhythmic complications, which consisted mainly of atrioventricular conductance delays or supraventricular tachycardias. Long-term follow-ups after a hospital discharge were only feasible in a small fraction of patients (36%, Table S1).

## 4. Discussion

The presented study investigated the relationship between the diastolic LAVV transvalvular pressure gradients, quantified using Doppler measurements, in intraoperative TEE studies after weaning from a CPB and postoperative TTE studies. As hypothesized, LAVV MPGs were significantly overestimated intraoperatively when compared to the awake postoperative examinations prior to discharge. The MPGs showed a linear correlation to a non-invasive surrogate of the CI, suggesting a dependency on the current hemodynamic condition after a CPB. Interestingly, this relationship was not observed in the awake TTE studies and most hemodynamic measures did not further differ between the two time-points. While the patients’ HRs differed significantly between the two examinations, no correlation between the LAVV transvalvular pressure gradients and the HRs were observed.

### 4.1. The Left Atrioventricular Valve following AVSD Repair

Depending on the various types of AVSDs, the LAVV often requires reconstruction during AVSD surgery [13]. LAVV reconstruction can range from simple annuloplasty to leaflet augmentation and chordal replacement [2]. Given the nature of the common atrioventricular valve, LAVV regurgitation is far more frequent after an AVSD repair than LAVV stenosis and represents the main cause of postoperative congestive heart failure with a need for reoperation [14,15]. Despite its rare occurrence, post-repair LAVV stenosis was shown to be associated with increased mortality [16]. A surgical revision is therefore indicated in the case of LAVV stenosis and, in some cases, requires an immediate re-initiation of a CBP within the primary intervention [17]. As AVSD repair is usually performed in young children, LAVV stenosis bears the risk of significantly worsening over time [9]. Thus, the diagnostic accuracy of LAVV stenosis quantification is crucial: while low sensitivities or underestimation of LAVV stenosis can lead to complicated postoperative courses and delayed reoperations, low specificities or overestimation of transvalvular diastolic pressure gradients expose the patients to the risks of unnecessary surgery and longer durations of CBPs [18,19].

### 4.2. Echocardiographic Modalities for the Assessment of LAVV Function

The choice of the acoustic window for an echocardiographic examination of the LAVV is mostly determined by the diagnostic time-point, the clinical environment and patient-specific factors, e.g., inaccessibility of the surgical site or insufficient transthoracic ultrasound quality. Whereas TTE is routinely used in awake patients outside the operating room, the TEE is performed intraoperatively or in sedated patients in the pediatric intensive care unit. The purpose of intraoperative TEE is mainly the detection of acute complications, the evaluation of the surgical result and the identification of additional surgical revision needed [20], while awake the TTE is primarily performed for preoperative AVSD characterization and the surveillance of long-term postoperative results [21]. In contrast to adult patients, cut-off values for the Doppler-based indices of LAVV or mitral valve inflow (MPG and PPG) have not been systematically validated in the pediatric population, let alone in AVSD patients [22]. While mild and moderate mitral valve stenosis in adults are separated by an MPG of 5 mmHg, the precise cut-off values remain elusive in pediatric patients after an AVSD repair and presumably require adjustment to the patients’ age [23]. Our data demonstrates that LAVV MPGs are significantly overestimated in early intraoperative TEE-based quantification, even if only to a moderate degree. Acute changes in the preload and afterload, associated with CPB and AVSD repair, should be taken into consideration during the interpretation of intraoperative TEEs, implicating a limited comparability with awake TTEs at later time-points. Under general anesthesia and after CPB, many factors may influence the hemodynamics and lead to a hyperdynamic circulatory state, such as inflammatory responses, resulting in tachycardia, decreased systemic vascular resistance and volume shifts. This may serve as a possible explanation for temporarily increased transvalvular pressure gradients but it cannot be concluded from our data. While data on LAVV stenosis are sparse due to its low postoperative incidence, previous studies report relevant discrepancies between intraoperative TEEs and awake TTEs regarding the detection of any relevant LAVV regurgitation [24,25,26]. A systematic difference between TTE- and TEE-derived Doppler gradients does thereby not appear to be a major factor for this phenomenon [27]. To our knowledge, our study is the first in the field to systematically investigate the impact of hemodynamic measures on the Doppler-derived transvalvular pressure gradients over the LAVV after an AVSD repair. 

### 4.3. Clinical Implications

While conducting an acute evaluation of the surgical result after weaning from a CPB using an intraoperative TEE, there is a fine line between confirming a successful AVSD repair and suggesting an immediate need for an additional surgical correction. While residual shunts are routinely quantified by color and pulsed-waved Doppler measurements, the reconstructed LAVV should be evaluated morphologically and functionally in multiple views [28]. To ensure a sufficient LAVV orifice, quantification of the mean inflow velocities using Doppler measurements yields reliable non-invasive transvalvular pressure gradients [29]. However, how helpful are these measurements for an acute intraoperative assessment of the LAVV after an AVSD repair? Our results might allow for two major clinical implications: (1) The current hemodynamic condition, reflected by the CI, seems to have a more severe impact on the MPG during intraoperative TEE when compared to awake TTE examinations; (2) Considering the potential overestimation of MPGs and the rare occurrence of relevant LAVV stenosis after an AVSD repair, MPGs, which might be regarded as concerningly elevated in preoperative or postoperative awake TEE examinations, seem more tolerable immediately after weaning from a CPB and other factors should be taken into account during an evaluation of LAVV function (e.g., direct Boulito probing by the surgeon or catheterization-based measurements). In our analysis, PPGs did not differ significantly between the two time-points. Regarding VTI morphology, MPGs may seem to be more prone to elevations due to increased hemodynamics than PPGs, and PPGs may better reflect actual LAVV stenosis. Prospective trials with appropriate scientific methodology are necessary to unveil the clinical applicability of our observations.

### 4.4. Limitations

The translation of our results to the general population is limited by the retrospective study design and the small number of included patients. Furthermore, patients with dramatically increased postoperative LAVV pressure gradients are underrepresented in our cohort. Due to the long study period, improvements in perioperative management and surgical techniques are possible and might lead to skewed observations. Owing to the retrospective data acquisition, many important measures were not systematically available–e.g., invasively quantified transvalvular gradients, invasively determined cardiac output or head-to-head comparisons of TTE- and TEE-derived parameters–which hinders the interpretation of the obtained results. The comparability of LV volumes and LVEF between TEE and TEE might be limited in pediatric patients. The surrogates of the CO and the CI used in this analysis are only simplified surrogates of the invasively determined parameters derived from gold-standard right-heart catheterization. Using only the LAVV orifice diameters acquired from a single plane might result in inadequate representation of the complex anatomy of the LAVV orifice area and lead to distinct variations between patients. While three-dimensional echocardiography is superior for the calculation of valve orifices, three-dimensional echocardiography data were not available in our retrospectively recruited cohort. Furthermore, three-dimensional transesophageal probes do not exist for neonates and infants. Lastly, long-term follow-up data was sparse.

## 5. Conclusions

Stenosis of the LAVV following the surgical repair of AVSD is a rare complication. In the presented investigation, the diastolic Doppler-quantified MPGs over the LAVV in an intraoperative TEE immediately after weaning from a CPB were significantly overestimated when compared to the gold-standard awake TTE prior to a hospital discharge. The LAVV MPGs showed a linear correlation to non-invasive surrogate measures of the CI. According to these findings, the current hemodynamic state should be taken into account during the interpretation of the LAAV gradients.

## Figures and Tables

**Table 1:** Baseline demographic and clinical characteristics of the study cohort.

Parameter	Result
Demographic data	
Age, days	151 (119–252)
Weight, kg	5.4 (4.7–6.2)
Height, cm	61 (58–64)
Body mass index, kg/m²	14.4 ± 1.5
BSA, m²	0.31 (0.28–0.33)
Morbidity	
AVSD	
Complete, n	34 (87)
Partial, n	4 (10)
Transitional, n	1 (3)
LAVV regurgitation, n	32 (82)
Right atrioventricular valve regurgitation, n	28 (72)
Additional congenital heart defects	
Patent Ductus Arteriosus, n	8 (21)
Pulmonary valve stenosis, n	4 (10)
Right ventricular outflow tract obstruction obstruction, n	2 (5)
Aortic stenosis, n	2 (5)
Coarctation, n	1 (3)
Borderline hypoplastic left heart syndrome, n	1 (3)
* Impaired left ventricular systolic function, n	4 (10)
* Impaired right ventricular systolic function, n	4 (10)
Symptoms of heart failure ^†^, n	32 (82)
Pulmonary hypertension ^‡^, n	20 (51)
Born preterm, n	7 (18)
Trisomy 21, n	25 (64)
Holt–Oram syndrome, n	1 (3)

Values are means ± standard deviations, medians (interquartile ranges) or n (%). AVSD = atrioventricular septal defect, BSA = body surface area, LAVV = left atrioventricular valve, * by visual estimation from awake pre-interventional echocardiograms, ^†^ cyanosis, diaphoresis during feeding, poor feeding and/or shortness of breath, ^‡^ mean pulmonary artery pressure > 25 mmHg preoperatively.

**Table 2:** Surgical and intraoperative data.

Parameter	Result
Surgical data	
AVSD repair	
Primary repair, n	38 (97)
Revision surgery, n	1 (3)
LAVV reconstruction, n	24 (62)
Right atrioventricular valve reconstruction, n	19 (49)
Pulmonary valve intervention, n	4 (10)
Patent Ductus Arteriosus closure, n	4 (10)
Right ventricular outflow tract myectomy, n	1 (3)
Aortic arch reconstruction, n	1 (3)
Duration of surgery, min	221 ± 56
Clamp time, min	88 ± 27
Reperfusion time, min	10 (7–16)
Residual LAVV regurgitation, n	34 (87)
Hemodynamics and mechanical ventilation during TEE	
MAP, mmHg	57 ± 11
CVP, mmHg	11 ± 4
PEEP, mbar	5 (5-5)
Mean airway pressure, mbar	9 (8–10)
Mean driving pressure, mbar	4 (3–5)
Vasopressors/inotropes	
Milrinone, mcg/kg/min	0.5 (0.3–0.5)
Norepinephrine, mcg/kg/min	0 (0–0.05)
Epinephrine, mcg/kg/min	0 (0–0)
VIS	6.0 (3.7–10.7)

Values are means ± standard deviations, medians (interquartile ranges) or n (%). AVSD = atrioventricular septal defect, CVP = central venous pressure, LAVV = left atrioventricular valve, MAP = mean arterial pressure, PEEP = positive end-expiratory pressure, TEE = transesophageal echocardiography, VIS = Vasoactive-Inotropic Score.

**Table 3:** Results of the echocardiographic measurements performed intraoperatively immediately after AVSD (TEE) repair and prior to discharge (TTE).

Parameter	Intraoperative	Discharge	*p*-Value
Heart rate, bpm	132 ± 17	114 ± 21	<0.001
LAVV MPG, mmHg	3.0 ± 1.2	2.3 ± 1.1	<0.01
LAVV PPG, mmHg	6.6 ± 2.7	5.7 ± 2.8	0.06
LAVV VTI, cm	16.1 ± 6.5	17.4 ± 6.6	0.18
Cardiac output, L/min	1.1 (0.8–1.7)	1.1 (0.8–1.5)	0.52
Cardiac index, L/min/m²	3.3 (2.4–5.1)	3.5 (2.5–4.3)	0.54
LVEF, %	44 ± 11	47 ± 13	0.16

Values are means ± standard deviations or (interquartile ranges). AVSD = atrioventricular septal defect, LAVV = left atrioventricular valve, LVEF = left ventricular ejection fraction, MPG = mean pressure gradient, PPG = peak pressure gradient, TEE = transesophageal echocardiography, TTE = transthoracic echocardiography, VTI = velocity-time integral.

**Table 4:** Postoperative data.

Parameter	Result
In-hospital	
Days between intraoperative TEE and discharge TTE	10 (6–15)
Duration of postoperative mechanical ventilation, h	70 (25–96)
Length of stay in intensive care unit, days	5 (3–9)
Length of stay in hospital, days	13 (10–17)
Adverse events	
None, n	21 (54)
Asystole / cardiopulmonary resuscitation, n	1 (3)
AVSD revision surgery, n	1 (3)
Intubation due to respiratory failure, n	1 (3)
Necrotizing enterocolitis, n	1 (3)
Persistent low cardiac output syndrome, n	1 (3)
Pulmonary bleeding, n	2 (5)
Pleural/pericardial effusion, n	5 (13)
Arrhythmias, n	13 (33)

Values are medians (interquartile ranges) or n (%). AVSD = atrioventricular septal defect, TEE = transesophageal echocardiography, TTE = transthoracic, echocardiography.

## Data Availability

Data can be obtained from the authors upon reasonable request in accordance with German privacy regulations.

## Supplementary Materials

The following supporting information can be downloaded at: https://www.mdpi.com/article/10.3390/diagnostics13050957/s1, Figure S1: Linear correlation matrices of echocardiographic, hemodynamic and ventilation parameters. Figure S2: Correlation diagrams of the cardiac index (CI) and heart rate (HR) with the mean pressure gradient (MPG) and the change of MPG between the two time-points (∆MPG); Table S1: Follow-up data.

## Abbreviations

AVSD	Atrioventricular septal defect
BSA	Body surface area
CI	Cardiac index
CPB	Cardiopulmonary bypass
CVP	Central venous pressure
HR	Heart rate
LAVV	Left atrioventricular valve
LVEF	Left ventricular ejection fraction
MAP	Mean arterial pressure
MPG	Mean pressure gradient
PPG	Peak pressure gradient
r	Correlation coefficient
TEE	Transesophageal echocardiography/echocardiogram
TTE	Transthoracic echocardiography/echocardiogram
VIS	Vasoactive-Inotropic Score
VTI	Velocity-time integral
