# Supplementary material for: Left Atrioventricular Transvalvular Pressure Gradients Derived from Intraoperative and Postoperative Echocardiograms following Atrioventricular Septal Defect Repair
Source: Diagnostics (Basel). 2023 Mar 2;13(5):957. doi: 10.3390/diagnostics13050957 (PMC10001237; doi:10.3390/diagnostics13050957)
Supplement: Supplementary file 1 [file diagnostics-13-00957-s001.zip › diagnostics-2234311-supplementary.pdf]

Supplementary Materials

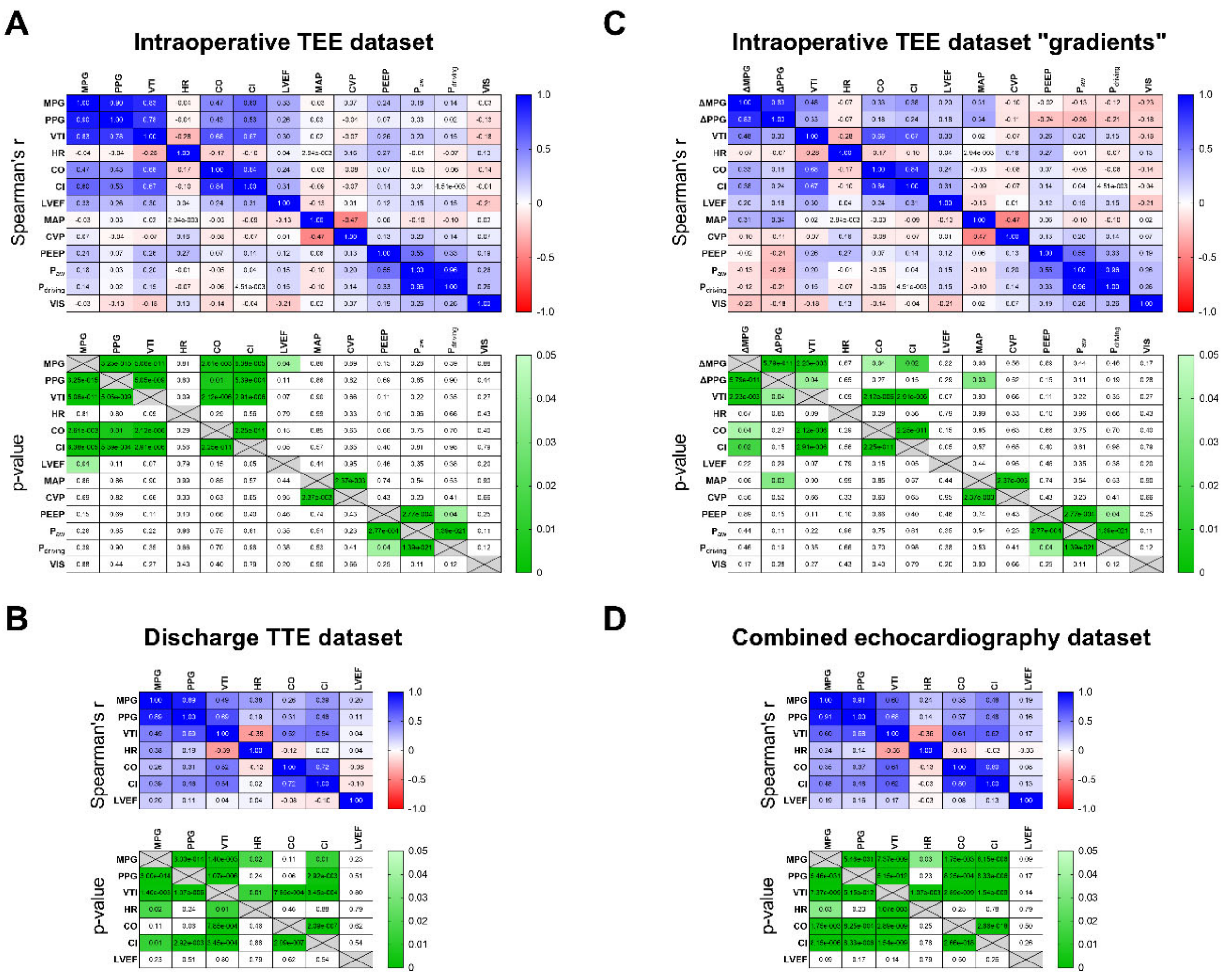

Figure S1: Linear correlation matrices of echocardiographic, hemodynamic and ventilation parameters

Spearman's correlation coefficients (r) and corresponding p-values between the recorded parameters at the time of intraoperative TEE (A) and pre-discharge TTE (B). Intraoperative measures were also correlated with the changes in mean pressure gradient (ΔMPG) and peak pressure gradient (ΔPPG) between the two echocardiograms (C). The correlation matrix of the combined dataset (intraoperative TEE and pre-discharge TTE) is shown under (D). CI = cardiac index, CO = cardiac output, CVP = central venous pressure, HR = heart rate, LVEF = left ventricular ejection fraction, MAP = mean arterial pressure, MPG =

mean pressure gradient, P_{aw} = mean airway pressure, $P_{driving}$ = driving airway pressure, PEEP = positive end-expiratory pressure, PPG = peak pressure gradient, VIS = Vasoactive-Inotropic score, VTI = velocity-time integral.

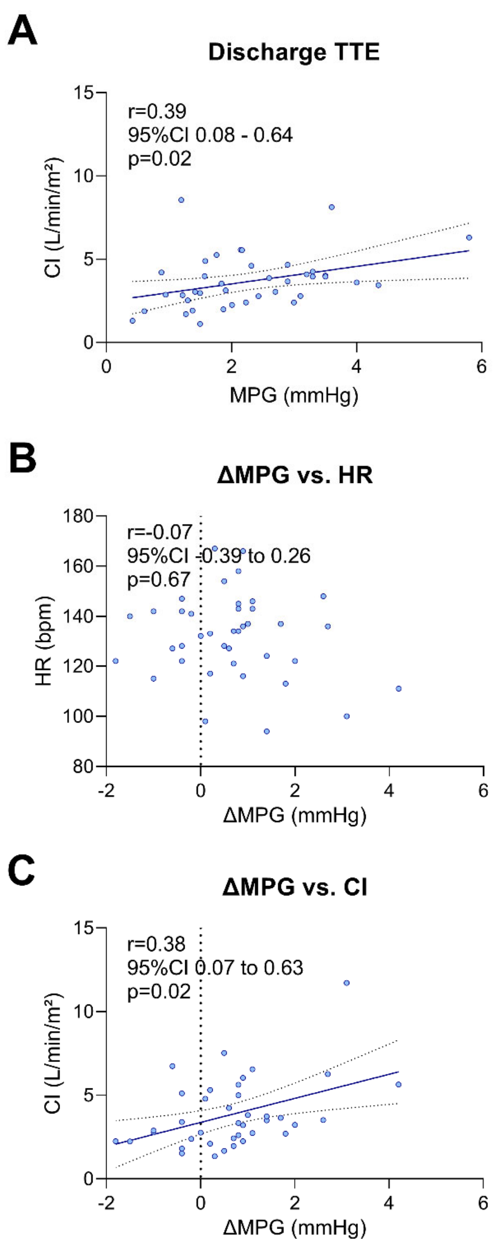

Figure S2: **Correlation diagrams of the cardiac index (CI) and heart rate (HR) with the mean pressure gradient (MPG) and the change of MPG between the two time-points (∆MPG)**

A weak linear correlation was observed between CI and MPG at the time of transthoracic echocardiography (TTE) prior to discharge (A). Correlation of ∆MPG with intraoperative HR (B) and CI (C) revealed no and a weak linear relationship, respectively. 95%CI = 95% confidence interval, r = Spearman's correlation coefficient.

Table S1: Follow-up data

Follow-up data	
Patients with post-discharge follow-up, n	14 (36)
Median follow-up time, d	789 (255-1,729)
Uneventful course, n	7 (50)
AVSD revision surgery, n	4 (29)
Portal vein thrombosis, n	1 (7)
Cardiopulmonary resuscitation due to seizure-induced hypoxia, n	1 (7)
Post-pericardiotomy syndrome, n	1 (7)
Good physical capacity, n	11 (79)

Values are medians (interquartile ranges) or n (%).

AVSD = atrioventricular septal defect
